# ChMinMaxPat: Investigations on Violence and Stress Detection Using EEG Signals

**DOI:** 10.3390/diagnostics14232666

**Published:** 2024-11-26

**Authors:** Omer Bektas, Serkan Kirik, Irem Tasci, Rena Hajiyeva, Emrah Aydemir, Sengul Dogan, Turker Tuncer

**Affiliations:** 1 Department of Pediatrics, Division of Pediatric Neurology, Faculty of Medicine, Ankara University, Ankara 06100, Turkey; bektasomer@gmail.com; 2 Department of Pediatrics, Division of Pediatric Neurology, Fethi Sekin City Hospital, Elazig 23280, Turkey; serkankirk34@gmail.com; 3 Department of Neurology, Firat University Hospital, Firat University, Elazig 23119, Turkey; itasci@firat.edu.tr; 4 Department of Information Technologies, Western Caspian University, Baku 1001, Azerbaijan; rena.haciyeva@wcu.edu.az; 5 Department of Management Information Systems, Management Faculty, Sakarya University, Sakarya 54050, Turkey; emrahaydemir@sakarya.edu.tr; 6 Department of Digital Forensics Engineering, Technology Faculty, Firat University, Elazig 23119, Turkey; turkertuncer@firat.edu.tr

**Keywords:** channel-based minimum and maximum pattern, Directed Lobish, EEG violence detection, EEG stress detection, explainable feature engineering, human forensics

## Abstract

Background and Objectives: Electroencephalography (EEG) signals, often termed the letters of the brain, are one of the most cost-effective methods for gathering valuable information about brain activity. This study presents a new explainable feature engineering (XFE) model designed to classify EEG data for violence detection. The primary objective is to assess the classification capability of the proposed XFE model, which uses a next-generation feature extractor, and to obtain interpretable findings for EEG-based violence and stress detection. Materials and Methods: In this research, two distinct EEG signal datasets were used to obtain classification and explainable results. The recommended XFE model utilizes a channel-based minimum and maximum pattern (ChMinMaxPat) feature extraction function, which generates 15 distinct feature vectors from EEG data. Cumulative weight-based neighborhood component analysis (CWNCA) is employed to select the most informative features from these vectors. Classification is performed by applying an iterative and ensemble t-algorithm-based k-nearest neighbors (tkNN) classifier to each feature vector. Information fusion is achieved through iterative majority voting (IMV), which consolidates the 15 tkNN classification results. Finally, the Directed Lobish (DLob) symbolic language generates interpretable outputs by leveraging the identities of the selected features. Together, the tkNN classifier, IMV-based information fusion, and DLob-based explainable feature extraction transform the model into a self-organizing explainable feature engineering (SOXFE) framework. Results: The ChMinMaxPat-based model achieved over 70% accuracy on both datasets with leave-one-record-out (LORO) cross-validation (CV) and over 90% accuracy with 10-fold CV. For each dataset, 15 DLob strings were generated, providing explainable outputs based on these symbolic representations. Conclusions: The ChMinMaxPat-based SOXFE model demonstrates high classification accuracy and interpretability in detecting violence and stress from EEG signals. This model contributes to both feature engineering and neuroscience by enabling explainable EEG classification, underscoring the potential importance of EEG analysis in clinical and forensic applications.

## 1. Introduction

Violence and stress detection is mainly concerned with understanding brain activity and physiological responses in high-pressure situations [1,2], and today, people are frequently faced with these situations for many different reasons, such as workload, educational activities, and traffic [3]. Using tools such as EEG signals, physiological data, or even video and audio inputs, identifying patterns in human behavior and brain function that may indicate violent acts or stress responses is an important area of research today [4,5]. By analyzing how the brain and body respond to such stimuli, researchers can develop real-time models that accurately detect and predict violence or stress [6]. This can be achieved through various methods, such as analyzing brain signals, heart rate, or even patterns in speech and facial expressions [7,8]. These developments are important for applications in public safety, mental health, and emergency response systems [9]. With the help of modern machine learning (ML) and deep learning (DL) models, the detection of these responses has become more precise [10]. For example, EEG data are increasingly used to capture brain activity reflecting stress or violent impulses [11]. Similarly, video and audio analysis can reveal aggressive acts or increased stress levels in real time [12]. These systems are being developed to operate with high accuracy, offering the potential for integration into health and safety environments [13]. By automating the detection of stress and violence, these tools not only help with timely intervention but also contribute to a deeper understanding of how the brain and body respond to high-stress situations [14].

### 1.1. The Literature Review

Some of the current studies on stress and violence detection in the literature are shown as follows. AlArfaj et al. [15] developed a model to detect violence-induced stress in Arabic social media posts using over 200 million Facebook posts. Their model attained an accuracy of 92.52%. Partila et al. [16] investigated stress detection from human speech in emergency situations using a Czech speech database of 31 recordings. They applied a support vector machine and achieved 87.90% accuracy. Yange et al. [17] developed a convolutional neural network (CNN)-based system to detect violence among ranch animals, using a dataset of violent and non-violent images. Their models achieved 93% accuracy for horses and sheep, 89% for goats, and 84% for cattle. Shindhe et al. [18] suggested a real-time violence detection system using deep neural networks, with a dataset of 1400 punching, 1050 kicking, and 2050 non-violent images. Their model achieved 96.12% accuracy for kicking and 94% for punching. Shahbazi et al. [19] presented a CNN-based model for detecting early life stress using physiological signals from 53 pregnant women. Their model achieved 95.69% accuracy for 30 s signals and 92.35% for 10 s signals. Kumari et al. [20] suggested a hybrid model for detecting cyber-aggressive posts using multi-modal data of images and text. Using a dataset of 3600 posts, their model combined VGG-16 and a CNN, achieving a weighted F1-score of 74.00%. Jaafar et al. [21] developed a multimodal fusion model using deep neural networks to detect aggression in surveillance, utilizing audio, video, and text data, along with five meta-features. The model achieved a weighted accuracy of 95.34%. Rendón-Segador et al. [22] introduced ViolenceNet, an approach to violence detection in videos, using DenseNet-3D, multi-head self-attention, and bidirectional ConvLSTM. Trained on four datasets, their model achieved up to 100% accuracy on simpler datasets and 96.90% on more complex ones. Anwar et al. [23] developed DeepSafety, a multimodal fusion model for detecting violence in conversations using 1295 audio segments. Their model achieved an F1-score of 85.00%. Singh et al. [24] introduced a video vision transformer for detecting violence in surveillance footage. They trained their model on datasets such as Hockey Fights and Violent Crowd, achieving high accuracy rates of 97.14% and 98.46%, respectively.

### 1.2. Literature Gaps

- Violence is an event that deeply affects the brain [25,26]. Therefore, the brain’s reaction to violence should be detected [25]. For this purpose, we used an EEG violence detection dataset to extract the brain’s reactions to violence. In the literature, various EEG signal classification methods exist, but there is a lack of research on EEG-based violence detection.
- Most of the EEG signal classification models have focused on classification accuracy, and there are fewer explainable artificial intelligence (XAI) models [27,28,29].
- Most of the EEG signal classification models have used a single dataset [30].
- To our knowledge, there has been no feature engineering research aiming to discover the differences in brain activation between violence and stress [31].

### 1.3. Motivation and Our Model

The main motivation of this research is to fill the existing gaps in the literature and demonstrate the effectiveness of statistical moments in feature extraction. Therefore, we have presented a new FE function, termed the channel-based minimum and maximum pattern (ChMinMaxPat). By using the recommended ChMinMaxPat FE function and the Directed Lobish (DLob) symbolic language, a next-generation self-organized explainable FE (SOXFE) model has been introduced. The presented SOXFE model consists of five main phases: (i) ChMinMaxPat-based FE, (ii) feature selection (FS) using cumulative weighted neighborhood component analysis (CWNCA), (iii) classification with t-algorithm-based k-nearest neighbors (tkNN) [32], (iv) information fusion through iterative majority voting (IMV) [33], and (v) generation of explainable results utilizing DLob.

The proposed ChMinMaxPat-based SOXFE model was tested on EEG violence detection and EEG stress detection datasets, each containing two classes: (i) violence/stress and (ii) control. By applying our model to the EEG violence detection dataset, we obtained both classification results and interpretable insights related to violence. Moreover, we created a connectome diagram representing the brain’s reaction to violence. Additionally, the proposed model has the capability to discover differences in brain activation between violence and stress by deploying DLob, providing further insights into how the brain responds to these distinct stimuli.

### 1.4. Novelties and Contributions

Novelties:
  - A new channel-based FE function, ChMinMaxPat, has been introduced.
  - An SOXFE model has been presented by deploying the proposed ChMinMaxPat, and this SOXFE model has been applied to EEG signal datasets for violence and stress detection.
  - We have used two EEG signal datasets, specifically for violence and stress detection. By using these datasets for testing, the overall high classification ability of the presented model has been demonstrated.

Contributions:
  - The presented ChMinMaxPat-based model achieved over 99% classification accuracy using both 10-fold and leave-one-record-out (LORO) cross-validation (CV). In this regard, we have introduced a highly accurate feature engineering model, contributing to the field of feature engineering.
  - We extracted interpretable results by deploying the DLob symbolic language, and using the generated DLob string, a connectome diagram related to violence was created.

## 2. Materials and Methods

### 2.1. Material

In this research, we used two EEG signal datasets, which were (i) EEG signals for violence detection and (ii) EEG signals for stress detection. For the violence detection dataset, we showed violent videos to participants. For the second dataset, we showed earthquake videos to survivors of the 2023 Great Turkey Earthquake Series. To collect these EEG datasets, the Emotiv Epoch X, a 14-channel brain cap (channels: 1: AF3; 2: F7; 3: F3; 4: FC5; 5: T7; 6: P7; 7: O1; 8: O2; 9: P8; 10: T8; 11: FC6; 12: F4; 13: F8; and 14: AF4), was used, following the 10/20 channel replacement system for EEG. The sampling frequency of the device is 128 Hz. Using this brain cap, EEG signals were collected from the frontal, temporal, parietal, and occipital lobes. For both datasets, EEG segments with a length of 15 s were used to create the EEG observations.

**Figure 1:**
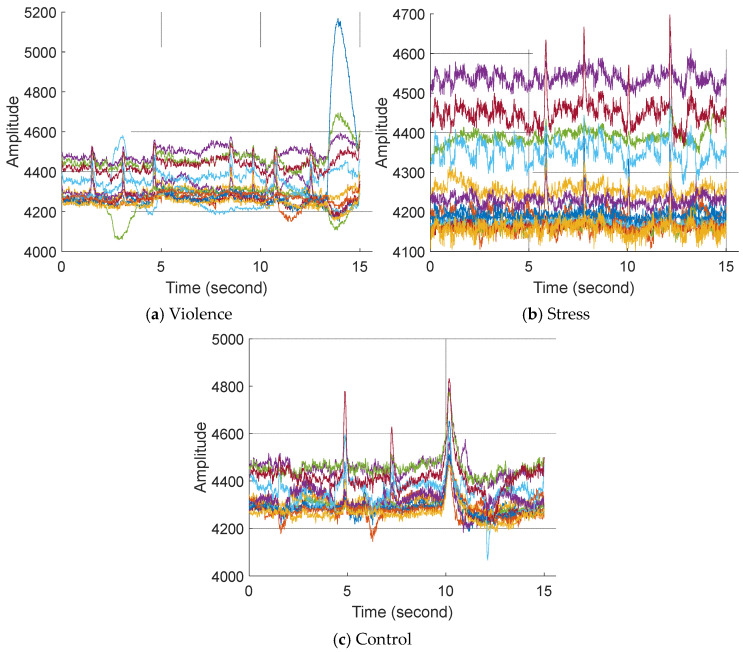
Sample EEG signals with 14 channels. Here, each color defines each channel.

A sample of the collected EEG signals with 14 channels, categorized by class, is demonstrated in Figure 1.

#### 2.1.1. The EEG Violence Detection Dataset

To the best of our knowledge, there are limited brain-based violence detection applications in the literature. However, violence is prevalent everywhere. To contribute to humanity and extract brain-based evidence of violence, an EEG violence detection dataset was used. Regarding the use of institutional oversight, the study protocol focused on ethical considerations, and all videos were carefully selected to minimize the participants’ distress. The video materials selected were pre-screened to exclude excessively disturbing content, and participants were fully informed about the nature of the study. In the information forms, we also stated that participants were free to stop providing EEG signals if they felt uncomfortable. This dataset was collected from 14 Turkish volunteers, all students, aged between 18 and 25, and there were 4 female and 10 male participants. Violence and meditation videos were shown to the participants during EEG signal collection, and the distribution of this dataset is presented in Table 1.

#### 2.1.2. The EEG Stress Detection Dataset

The major objective of the EEG stress detection dataset was to detect earthquake-related stress responses using EEG signals. The participants in this dataset were survivors affected by the Great Turkey Earthquake Series on 6 February 2023. To evoke earthquake-related stress, real earthquake footage was shown to the participants, while relaxing videos were displayed to the control group. This dataset was collected from 310 Turkish participants, aged 18 to 63, consisting of 42 women and 268 men. Based on their self-reported stress levels, 150 participants were labeled as stressed, while 160 were labeled as controls. The distribution of this dataset is shown in Table 2. In this dataset, each record belongs to a specific subject.

### 2.2. Method

The major objective of the proposed ChMinMaxPat-based model is to achieve high classification performance with interpretable results.

Nowadays, in the literature, most researchers use deep learning (DL) models to achieve high classification performance. However, DL models utilize millions of parameters, resulting in very high time complexities. Feature engineering models, on the other hand, have relatively lower classification accuracies but exhibit linear time complexity. Therefore, we aimed to address this trade-off by presenting a new self-organized feature engineering model.

The proposed ChMinMaxPat-based model consists of five main phases:

- ChMinMaxPat-based feature extraction (FE);
- CWNCA-based feature selection;
- Classification utilizing tkNN;
- IMV-based information fusion;
- Generation of explainable results with DLob.

**Figure 2:**
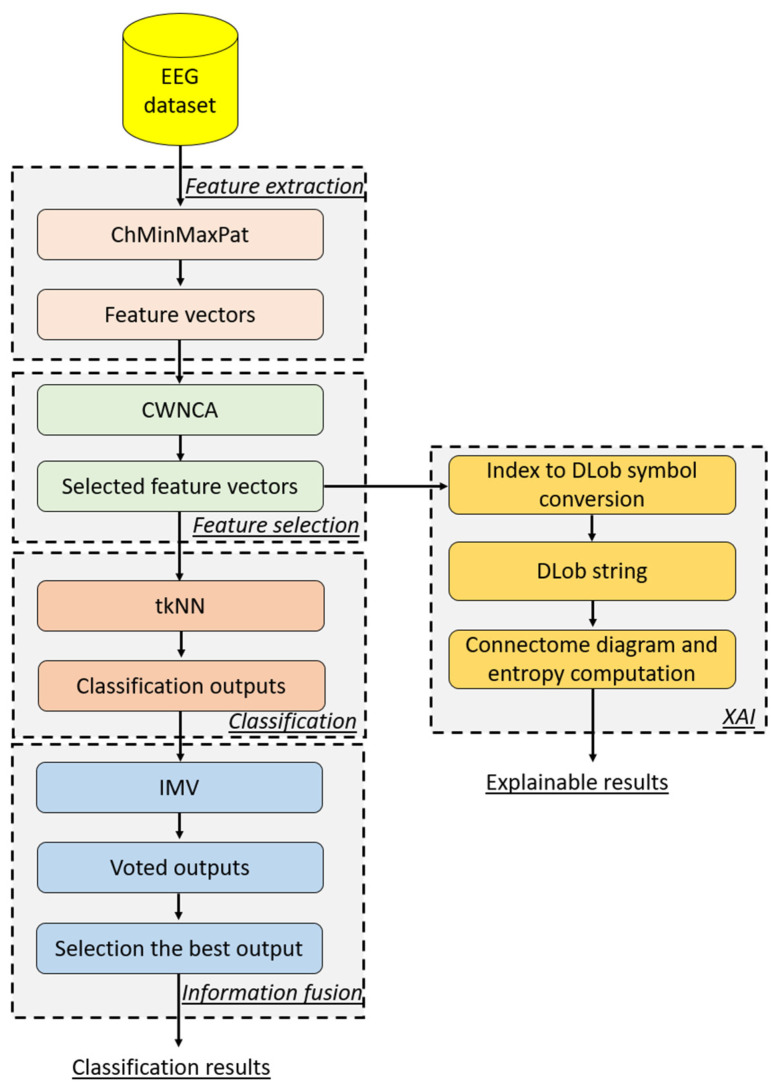
A graphical overview of the presented ChMinMaxPat-based SOXFE model.

To explain the proposed ChMinMaxPat-based model better, a graphical depiction of this model is shown in Figure 2.

As can be seen from Figure 2, the introduced SOXFE model generated 15 feature vectors by deploying the recommended ChMinMaxPat feature extraction function. In the feature selection phase, the CWNCA feature selector was applied to the 15 feature vectors generated, resulting in 15 selected feature vectors. These selected feature vectors were used as the input to the tkNN classifier, and the indices of these feature vectors were used to create 15 DLob strings. By utilizing these 15 DLob strings, 15 connectome diagrams were created. In the classification phase, tkNN generated 15 classifier-based outcomes. In the information fusion phase, IMV was applied to the 15 classifier-based outcomes generated, and IMV produced 13 voted outcomes from these 15 classifier-based outcomes. The best outcome (the voted outcome with the highest classification accuracy) was selected using a greedy algorithm [34]. In this regard, the presented model is both a self-organized and explainable feature engineering model.

The details of the recommended ChMinMaxPat-based model are provided below.

#### 2.2.1. The Proposed ChMinMaxPat-Based Feature Extraction

The first phase of this model is feature extraction, and ChMinMaxPat was utilized as the main FE function. Therefore, the steps of the ChMinMaxPat presented are demonstrated below.

Step 1: Read the channels of each EEG point and create a channel vector. In this research, both datasets used have 14 channels. Therefore, the length of each generated vector is 14.

(1) $$vec^{i}=EEG\left(i,1:nc\right),\;i\in \{1,2,\ldots ,L\}$$

where vec: a vector; EEG: the EEG signal; nc: the number of channels; and L: the length of the signal.

Step 2: Compute the distance of the vector from the average value of the vector.

(2) $$dist^{i}=\left|vec^{i}-av^{i}\right|$$

(3) $$av^{i}=\frac{1}{nc}\sum_{j=1}^{nc}vec^{i}\left(j\right)$$

where dist is the distance of the vector values and av is the average value.

Step 3: Calculate the minimum and maximum indices of the channel values and their corresponding distances.

(4) $$id_{1}^{i}=argmax\left(vec^{i}\right)$$

(5) $$id_{2}^{i}=argmin\left(vec^{i}\right)$$

(6) $$id_{3}^{i}=argmax\left(dist^{i}\right)$$

(7) $$id_{4}^{i}=argmin\left(dist^{i}\right)$$

where id is the identity of the minimum and maximum values.

Step 4: Fill the 6 transition tables by deploying the identity values. Here, a combinational feature generation method was utilized.

(8) $$tt_{k}^{i}=\left[\begin{array}{ccc} 0 & \cdots & 0\\ \vdots & \ddots & \vdots \\ 0 & \cdots & 0 \end{array}\right],\;k\in \left\{1,2,\ldots ,6\right\}$$

(9) $$tt_{1}\left(id_{1}^{i},id_{2}^{i}\right)=tt_{1}\left(id_{1}^{i},id_{2}^{i}\right)+1$$

(10) $$tt_{2}\left(id_{1}^{i},id_{3}^{i}\right)=tt_{2}\left(id_{1}^{i},id_{3}^{i}\right)+1$$

(11) $$tt_{3}\left(id_{1}^{i},id_{4}^{i}\right)=tt_{3}\left(id_{1}^{i},id_{4}^{i}\right)+1$$

(12) $$tt_{4}\left(id_{2}^{i},id_{3}^{i}\right)=tt_{4}\left(id_{2}^{i},id_{3}^{i}\right)+1$$

(13) $$tt_{5}\left(id_{2}^{i},id_{4}^{i}\right)=tt_{5}\left(id_{2}^{i},id_{4}^{i}\right)+1$$

(14) $$tt_{6}\left(id_{3}^{i},id_{4}^{i}\right)=tt_{6}\left(id_{3}^{i},id_{4}^{i}\right)+1$$

Herein, tt is the transition table.

Step 5: Calculate the feature map signals to generate histogram-based features.

(15) $$map_{1}\left(i\right)=\left(id_{1}^{i}-1\right)nc+id_{2}^{i}-1$$

(16) $$map_{2}\left(i\right)=\left(id_{1}^{i}-1\right)nc+id_{3}^{i}-1$$

(17) $$map_{3}\left(i\right)=\left(id_{1}^{i}-1\right)nc+id_{4}^{i}-1$$

(18) $$map_{4}\left(i\right)=\left(id_{2}^{i}-1\right)nc+id_{3}^{i}-1$$

(19) $$map_{5}\left(i\right)=\left(id_{2}^{i}-1\right)nc+id_{4}^{i}-1$$

(20) $$map_{6}\left(i\right)=\left(id_{3}^{i}-1\right)nc+id_{4}^{i}-1$$

Herein, map is the feature map signal.

Step 6: Repeat Steps 1–5 until the entire length of the EEG signal has been processed, and then generate transition tables and feature map signals.

Step 7: Repeat Steps 1–6 until the entire EEG signal has been processed, and then generate transition tables and feature map signals.

Step 8: Normalize the transition tables by dividing each row by the summation of its elements.

(21) $$tt_{k}^{i}=\frac{tt_{k}^{i}\left(a,b\right)}{\sum_{b=1}^{nc}tt_{k}^{i}\left(a,b\right)+\varepsilon },\;a,b\in \{1,2,\ldots ,nc\}$$

where ε is epsilon, a small value used to avoid division by zero errors.

Step 9: Apply matrix-to-vector transformation to the transition tables generated and obtain six feature vectors.

(22) $$fv_{k}\left(g\right)=tt_{k}\left(a,b\right),\;g\in \left\{1,2,\ldots ,nc^{2}\right\}$$

Step 10: Concatenate the six generated feature vectors (feature vectors 1–6) to create the seventh feature vector.

(23) $$fv_{7}\left(g+nc^{2}\left(k-1\right)\right)=fv_{k}\left(g\right)$$

Step 11: Extract histograms of the feature map signals and obtain an additional six feature vectors.

(24) $$fv_{7+k}=\theta \left(map_{k}\right)$$

Here, θ(.) is the histogram extraction function, and the length of the generated feature vector is $nc^{2}$.

Step 12: Merge the histogram-based feature vectors generated to obtain the 14th feature vector.

(25) $$fv_{14}\left(g+nc^{2}\left(k-1\right)\right)\;=fv_{7+k}\left(g\right)$$

Step 13: Create feature vector 15 by concatenating feature vectors 7 and 14, as these are the merged feature vectors. This step generates a general feature vector, and feature vector 15 represents the combination of the 14 previously generated feature vectors.

(26) $$fv_{15}\left(z\right)=fv_{7}\left(z\right),\;z\in \left\{1,2,\ldots ,6nc^{2}\right\}$$

(27) $$fv_{15}\left(6nc^{2}+z\right)\;=fv_{14}\left(z\right)$$

The 13 steps outlined above define the presented ChMinMaxPat feature extractor, and this feature extractor generates 15 feature vectors.

#### 2.2.2. Feature Selection

In the feature selection phase, 15 feature vectors were generated. To select the most informative features from these 15 feature vectors, the CWNCA feature selector was used. This feature selector is an improved version of the NCA [35] feature selector. In CWNCA, the number of selected feature vectors is determined by deploying cumulative weight computation. The steps of the CWNCA feature selector are demonstrated below.

Step 14: Compute the feature maps and the qualified indices of the features using the NCA feature selector.

(28) $$w_{h}=NCA\left(fv_{h},y\right),\;h\in \{1,2,\ldots ,15\}$$

(29) $$ind_{h}=argsort\left(-w_{h}\right)$$

where w: the weight of the features; NCA(.): the NCA feature selector; y: the real outcome; and ind: the qualified indices.

Step 15: Calculate the number of selected features by deploying cumulative weight computation. Here, the threshold value used is 0.99.

(30) $$nf_{h}=CW(fv_{h},w_{h},ind_{h},99)$$

where nf is the number of features, and CW(.) is the cumulative weight computation.

Step 16: Select the most informative features by using the computed indices and the number of features determined through cumulative weight computation.

(31) $$sf_{h}\left(d,x\right)=fv_{h}\left(d,ind_{h}\left(x\right)\right),\;x\in \left\{1,2,\ldots ,nf_{h}\right\},\;d\in \left\{1,2,\ldots ,N\right\}\;$$

where sf is the selected feature vector, and N is the number of the EEG signal.

The selected features were used in two phases: the classification phase and the XAI phase. In the classification phase, the selected feature vectors were used as input to the tkNN classifier. In the XAI phase, the identities of these feature vectors were utilized for DLob symbol selection.

#### 2.2.3. Classification

To generate the classification outcomes for the 15 selected feature vectors, the tkNN classifier was used, which is an iterative and ensemble version of the kNN classifier. In the tkNN classifier, the parameters of the kNN [36] classifier are changed iteratively, and one outcome is generated for each parameter. Afterward, voted outcomes are produced by utilizing these parameter-based outcomes. Subsequently, the best outcome is selected from the generated parameter-based and voted outcomes using a greedy algorithm.

Step 17: Generate the parameter-based outcomes by deploying iterative parameter changes in the kNN classifier.

(32) $$pot_{s}^{h}=kNN\left(sf_{h},y,K_{i},D_{j},Wg_{k}\right),\;s\in \left\{1,2,\ldots ,60\right\},\;K=\left\{1,2,\ldots ,10\right\},\;D=\left\{CityBlock,\;Euclidean,\;Cosine\right\},D=\{Inverse,\;Equal\}\;$$

Herein, pot: the parameter-based outcome; K: the k values of kNN; D: a distance metric; and Wg: weight. Moreover, 10-fold CV and LORO CV were utilized to generate these results.

Step 18: Create 58 (=60 − 3 + 1) voted outcomes by deploying the IMV algorithm. A mathematical representation of the IMV algorithm is shown below:

(33) $$acc\left(s\right)=\alpha \left(pot_{s}^{h},y\right)\;\;$$

(34) idx=argsort(−acc)

(35) $$vot_{r}^{h}=\varpi \left(pot_{idx\left(1\right)}^{h},pot_{idx\left(2\right)}^{h},\;\ldots ,pot_{idx\left(r+2\right)}^{h}\right),\;r\in \left\{1,2,\ldots ,58\right\}$$

Here, acc is the classification accuracy, and we sort the outcomes per the classification accuracy; α(.) is the function for calculating the classification accuracy; idx represents the qualified indexes of the parameter-based outcomes in descending order; and vot is the voted outcomes. Equations (33)–(35) define the IMV function.

Step 19: Choose the most accurate outcome as the final outcome from the 118 generated outcomes (60 parameter-based + 58 voted).

(36) $$acc\left(60+r\right)=\alpha \left({vot}_{r}^{h},y\right)\;\;$$

(37) index=argmax(acc)

(38) $$fout^{h}=\left\{\begin{array}{c} pot_{index}^{h},\;index\leq 60\\ vot_{index-60}^{h},index>60 \end{array}\right.$$

where index is the index of the maximum accurate outcome, and fout is the selected final outcome. Equations (36)–(38) define the greedy algorithm.

In this phase, 15 tkNN-based outcomes were generated.

#### 2.2.4. Information Fusion

The main objective of this phase is to improve the classification performance of the tkNN-based outcomes. Therefore, we applied IMV to generate 13 (=15 − 3 + 1) voted outcomes from the 15 tkNN-based outcomes. Using a greedy algorithm, the best outcome was selected from these 13 voted outcomes. The steps of this phase are as follows:

Step 20: Create 13 voted outcomes from the 15 tkNN-based outcomes by applying the IMV algorithm.

(39) vot=IMV(pout)

where IMV(.) is the IMV algorithm.

Step 21: Select the best outcome by utilizing the greedy algorithm.

(40) final=GA(vot)

Herein, final is the final outcome, and GA(.) is the greedy algorithm.

By utilizing this phase, the final classification outcome was generated, and the classification results of the presented ChMinMaxPat-based model were produced.

#### 2.2.5. Generation of Explainable Results

The last phase is the XAI phase. In this phase, DLob was integrated into the presented ChMinMaxPat-based model. DLob generates symbols based on the channels used. In this research, two datasets were utilized, each with 14 channels. The DLob symbols used and their meanings are shown in Table 3.

By using the channel information, the DLob symbols were extracted, and each feature vector contained two DLob symbols. By utilizing the identities/indexes of the selected features, the DLob symbols were generated, and these DLob symbols were concatenated to create DLob strings. In this phase, by deploying the generated 15 DLob strings, information entropies and transitions of the symbols were created to present interpretable results of the recommended model. The steps of this presented XAI phase are as follows:

Step 22: Generate the DLob strings by deploying the indexes of the selected feature vectors.

(41) $$value_{h}\left(x\right)=ind_{h}\left(x\right),\;x\in \left\{1,2,\ldots ,nf_{h}\right\}\;\;$$

(42) $$mul=\left\lfloor \frac{value_{h}\left(x\right)-1}{nc^{2}}\right\rfloor$$

(43) $$rem=value_{h}\left(x\right)-nc^{2}\times mul$$

(44) $$mul=\left\lfloor \frac{value_{h}\left(x\right)-1}{nc^{2}}\right\rfloor$$

(45) $$first=\left\lfloor \frac{rem-1}{nc}\right\rfloor +1$$

(46) $$second=\left[rem-1\;\left(mod\;nc\right)\right]+1$$

(47) LUT={FL,FL,FL,FL,TL,PL,OL,OR,PR,TR,FR,FR,FR,FR}

(48) $$str_{h}\left(c\right)=LUT\left(first\right),\;c\in \left\{1,3,\ldots ,2nf_{h}-1\right\}$$

(49) $$str_{h}\left(c+1\right)=LUT\left(second\right)$$

where value: the index of the selected feature; mul: the multiplication coefficient since we used concatenated feature vectors; rem: the remainder value,; first: the index of the first DLob symbol; second: the index value of the second DLob symbol since each feature contains two pieces of DLob symbol information; LUT: a look-up-table of the used brain cap; and str: the DLob string created. By deploying this step, 15 DLob strings.

Step 23: Compute the information entropy of each generated DLob string.

(50) $$histo_{h}=\theta (str_{h})\;\;$$

(51) $$pr_{j}^{h}=\frac{histo_{h}(j)}{\sum_{j=1}^{8}histo_{h}(j)}$$

(52) $$ent_{h}=-\sum_{j=1}^{8}pr_{j}^{h}\log(pr_{j}^{h})$$

Herein, histo is a histogram of the symbols, and we used 8 symbols in this research; pr is the probability value; and ent is the entropy value of the corresponding DLob string.

Step 24: Calculate the transitions of the symbols and create connectome diagrams.

This phase is the final phase of the proposed ChMinMaxPat-based SOXFE model, and through this phase, interpretable results were generated. Moreover, the 24 steps outlined above define the introduced ChMinMaxPat-based SOXFE model.

## 3. Results

In this research, we presented a new SOXFE model. The entire model was coded in the MATLAB (version 2024a) programming environment. To create these phases, custom functions were developed. For instance, the ChMinMaxPat function was created to implement feature extraction. Moreover, the proposed ChMinMaxPat-based SOXFE model is a feature engineering model with linear time complexity. Therefore, a simple configured computer was utilized for its implementation. This computer has a 3.2-gigahertz central processing unit (CPU) and 32 gigabytes of main memory. No parallel programming, processing methods, or devices were used. The ChMinMaxPat-based model presented was implemented using CPU mode.

This model uses the ChMinMaxPat, CWNCA, tkNN, IMV, greedy algorithm, and XAI generation functions. These functions are parametric, and the parameters used in these functions are tabulated in Table 4.

By using the parameters provided above (see Table 4), the proposed ChMinMaxPat-based SOXFE model was implemented, and our approach generated both classification and explainable results.

### 3.1. Classification Results

The first results generated by the recommended ChMinMaxPat-based SOXFE model (the EEG classification model) are the classification results. In order to obtain classification results, commonly used classification performance metrics were used, and these performance metrics were (1) classification accuracy (acc), (2) sensitivity (sen), (3) specificity (spe), and (4) geometric mean (gm). These performance metrics were computed using the number of true positives (tp), false negatives (fn), true negatives (tn), and false positives (fp). The mathematical expressions of these performance metrics are depicted below.

(53) $$acc=\frac{tp+tn}{tp+fn+fp+tn}\;$$

(54) $$sen=\frac{tp}{tp+fn}$$

(55) $$spe=\frac{tn}{fp+tn}$$

(56) $$gm=\sqrt{\frac{tp}{tp+fn}\times \frac{tn}{fp+tn}}$$

**Figure 3:**
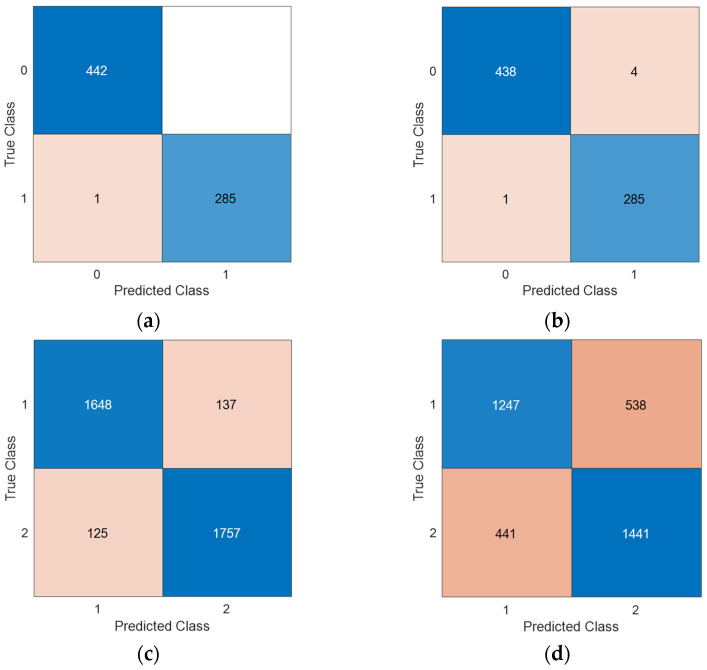
Confusion matrices of the datasets used. (i) The EEG violence detection dataset. Herein, 0 is the control, and 1 is violence; (**a**) 10-fold CV; (b) LORO CV. (ii) The EEG stress detection dataset. Herein, 1 is stress, and 2 is the control; (**c**) 10-fold CV; (**d**) LORO CV.

Moreover, we used two validation techniques, and these were (i) 10-fold CV and (ii) LORO CV. In order to compute the four performance evaluation metrics used, the confusion matrices generated by deploying the ChMinMaxPat-based SOXFE introduced are demonstrated in Figure 3.

According to Figure 3, the results computed are listed in Table 5.

In the EEG stress detection dataset, each record belongs to a unique participant; therefore, this LORO CV can be referred to as leave-one subject-out (LOSO) CV.

For the EEG violence detection dataset, all the results exceeded 99%, but this dataset is relatively small. In the EEG stress detection dataset, classification accuracies of 92.86% and 73.30% were obtained using 10-fold CV and LOSO CV, respectively.

The performance differences between 10-fold CV and LORO/LOSO CV indicate the impact of the data composition on the model’s accuracy. In 10-fold CV, data from the same participant can appear in both training and testing, allowing the model to learn individual-specific patterns, which results in higher accuracy. In contrast, LORO/LOSO CV excludes all data from one participant/recording in each test iteration, providing a more stringent test of the model’s ability to generalize across individuals/recordings, leading to more reliable results. This discrepancy is minimal for the smaller, more homogeneous EEG violence dataset. However, for the more variable EEG stress dataset, LOSO CV results in a significantly lower accuracy (73.30%), highlighting the difficulty of generalizing across stress-related responses. This also clearly showcases that stress can originate from different endogenous sources.

### 3.2. Explainable Results

Our proposal is an SOXFE model, and therefore, it generates explainable results. In this model, for each dataset, 15 DLob strings were generated. These 15 DLob strings were used to create connectome diagrams and calculate entropy values. The DLob strings generated for the EEG violence detection and EEG stress detection models are shown in Appendix A Table A1 and Table A2.

**Figure 4:**
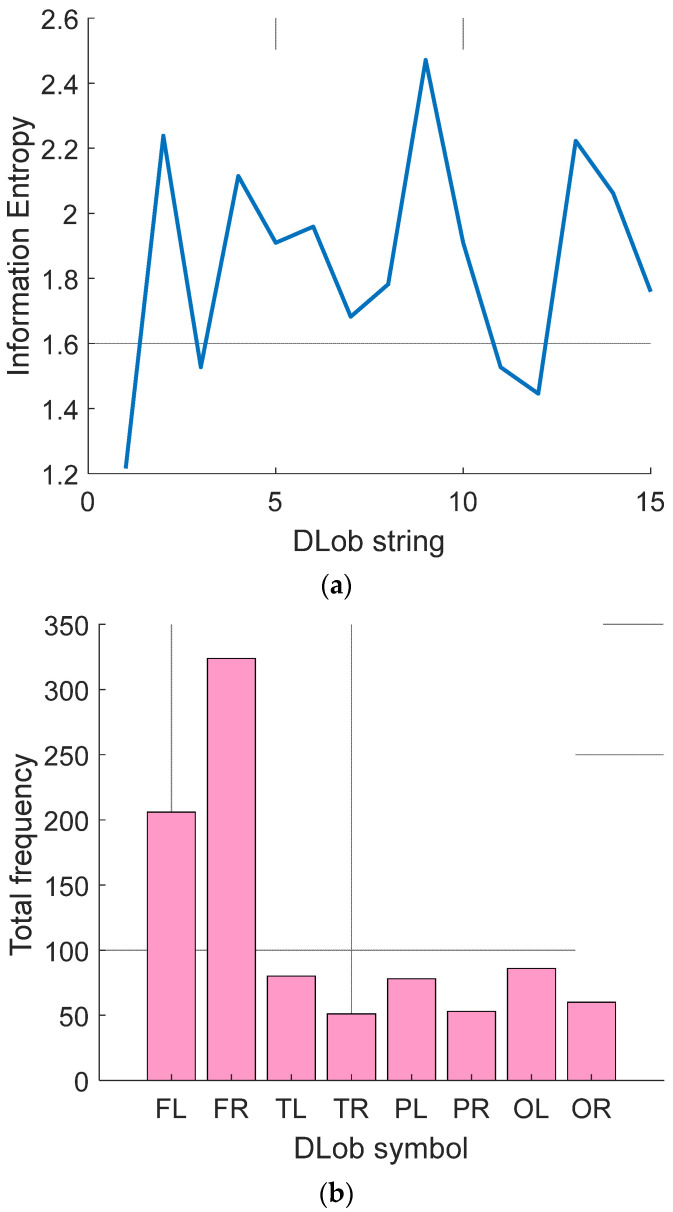
Analysis of the violence detection DLob strings. (**a**) Entropy; (**b**) Histogram.

Table A1 demonstrates the generated DLob strings for violence detection. The information entropies of these strings were computed, and these entropy values, along with the average usage frequencies of the symbols, are showcased in Figure 4.

According to Figure 4a, the information entropies computed range from 1.2164 to 2.4711. In this research, eight DLob symbols were used, so the maximum possible information entropy is 3 (${=\log}_{2}8$). This indicates that the DLob strings generated related to violence detection are relatively predictable, and a rule-based decision support model for violence detection can be proposed.

As shown in Figure 4b, the most frequently used lobe is the frontal lobe. These results clearly demonstrate that the brain region most affected by violence is the frontal lobe.

The second dataset is the EEG stress detection dataset, which focused on earthquake stress, and the DLob symbols generated are shown in Table A2.

**Figure 5:**
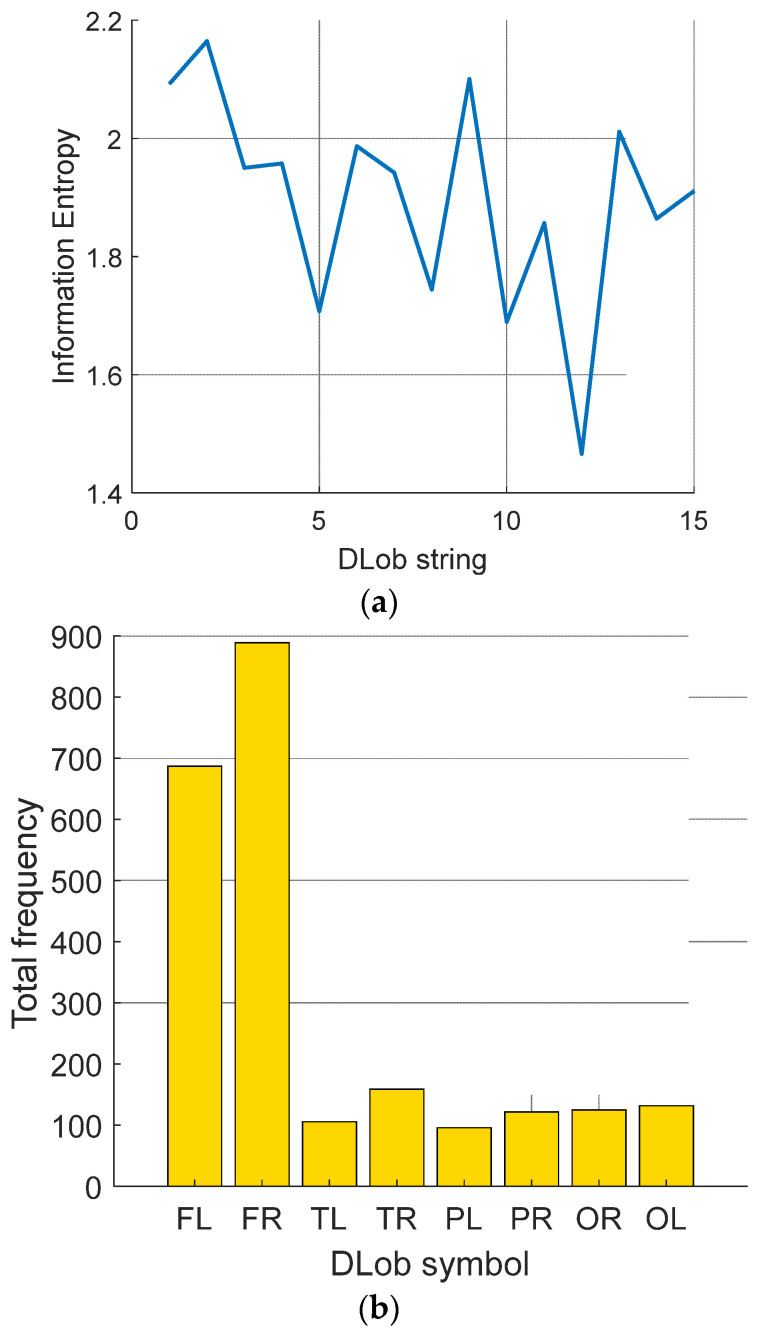
Analysis of the stress detection DLob strings. (**a**) Entropy; (**b**) Histogram.

Table A2 showcases the DLob strings generated for stress detection. The information entropies of these strings were computed, and these entropy values, along with the average usage frequencies of the symbols, are presented in Figure 5.

According to Figure 5a, the information entropies computed range from 1.4658 to 2.1646. The DLob strings generated for stress detection are predictable, suggesting that a rule-based decision support model for stress detection can be proposed.

As shown in Figure 5b, the most frequently used lobe is the frontal lobe, which is expected since this dataset relates to earthquake stress.

**Figure A1:**
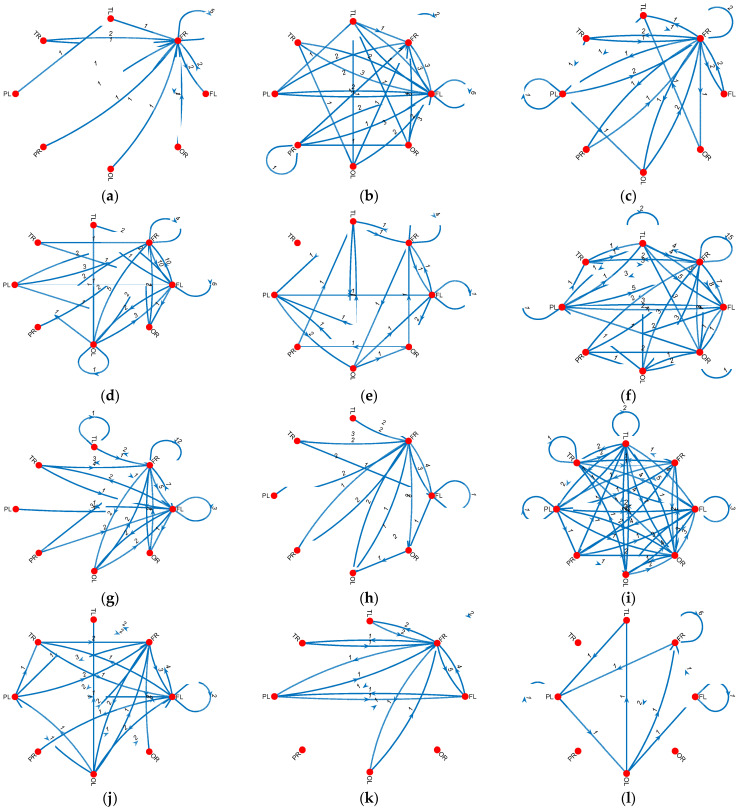

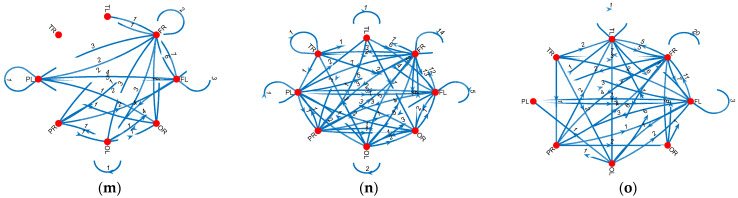
Connectome diagrams from EEG violence detection; (**a**) Selected feature vector 1; (**b**) Selected feature vector 2; (**c**) Selected feature vector 3; (**d**) Selected feature vector 4; (**e**) Selected feature vector 5; (**f**) Selected feature vector 6; (**g**) Selected feature vector 7; (**h**) Selected feature vector 8; (**i**) Selected feature vector 9; (**j**) Selected feature vector 10; (**k**) Selected feature vector 11; (**l**) Selected feature vector 12; (**m**) Selected feature vector 13; (**n**) Selected feature vector 14; (**o**) Selected feature vector 15.

**Figure A2:**
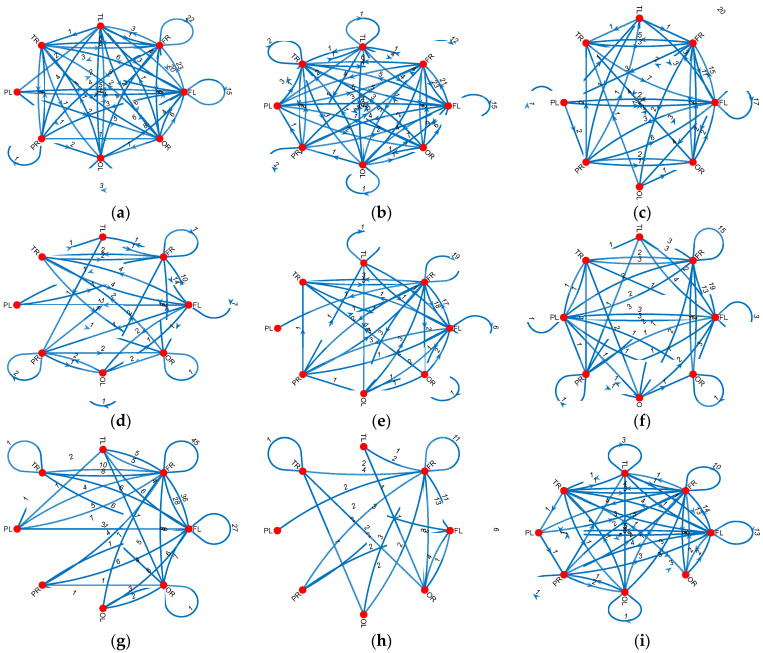

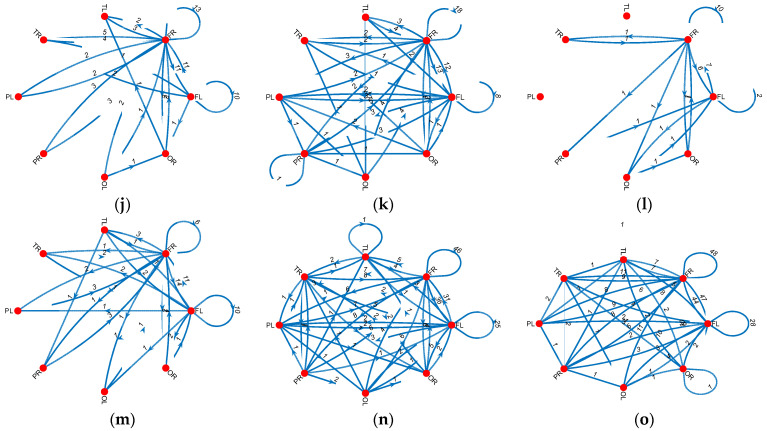
Connectome diagrams from EEG stress detection. (**a**) Selected feature vector 1; (**b**) Selected feature vector 2; (**c**) Selected feature vector 3; (**d**) Selected feature vector 4; (**e**) Selected feature vector 5; (**f**) Selected feature vector 6; (**g**) Selected feature vector 7; (**h**) Selected feature vector 8; (**i**) Selected feature vector 9; (**j**) Selected feature vector 10; (**k**) Selected feature vector 11; (**l**) Selected feature vector 12; (**m**) Selected feature vector 13; (**n**) Selected feature vector 14; (**o**) Selected feature vector 15.

Additionally, the connectome diagrams of these DLob strings are showcased in Appendix A Figure A1 and Figure A2.

## 4. Discussion

The proposed ChMinMaxPat-based model generates both classification and explainable results for the datasets used. Our proposal achieved over 99% classification accuracy for violence detection and over 70% accuracy for stress detection. The recommended ChMinMaxPat-based SOXFE model uses two self-organized phases: classification and information fusion. In the classification phase, the tkNN classifier was employed, which selected the best outcome from the 118 created outcomes. The IMV-based information fusion phase generates 13 voted outcomes from the 15 tkNN-based outcomes and automatically selects the best final outcome. As a result, the presented model achieved high classification performance.

**Figure 6:**
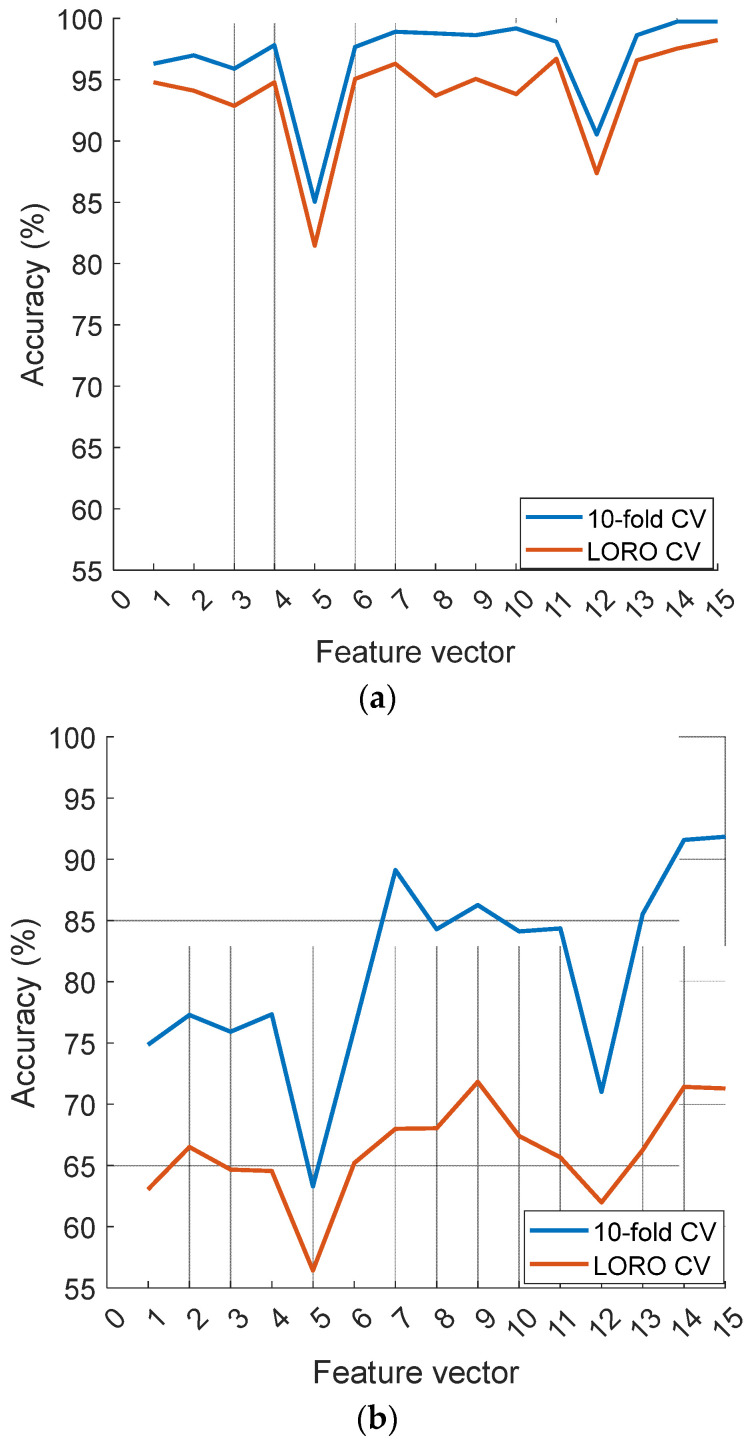
Comparison of the feature vectors’ classification abilities. (**a**) Violence detection; (**b**) Stress detection.

To generate these 15 tkNN-based classification results, 15 selected feature vectors were used, and these feature vectors were generated by the recommended ChMinMaxPat method. The classification performance of these feature vectors is compared in Figure 6.

Figure 6 clearly demonstrates that the 5th feature vector is the least accurate for both datasets, while the concatenated feature vectors (7, 14, and 15) perform better than the other feature vectors. Furthermore, feature vectors 1–6 were generated by deploying the transition table, and feature vectors 8–13 were created using channel coding and histogram extraction. According to Figure 6, the channel-coding- and histogram-extraction-based feature vectors exhibit a higher classification performance than that of the transition-table-based feature vectors.

**Figure 7:**
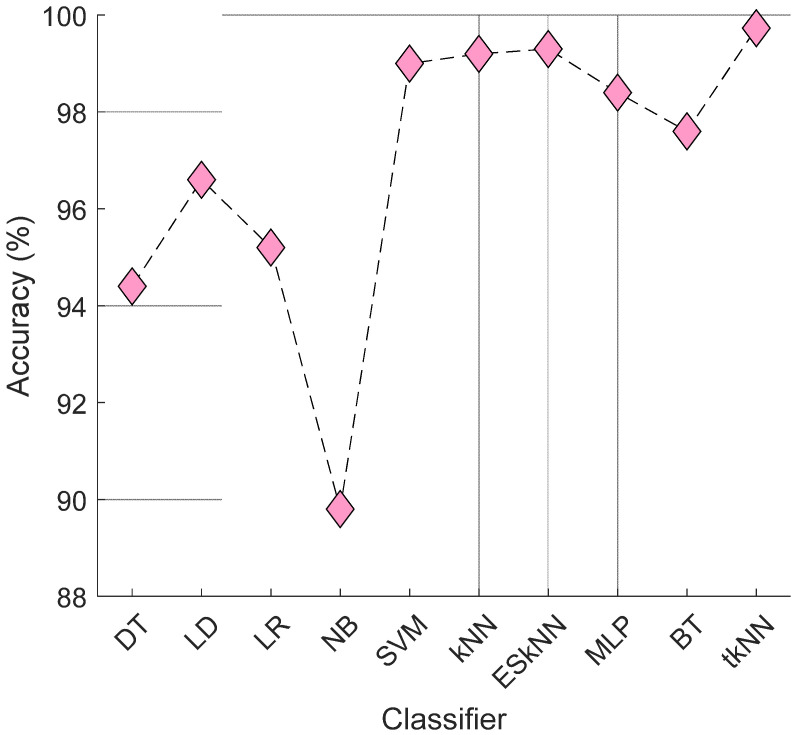
A performance comparison of the classifiers using the 14th feature of the EEG violence detection dataset. Herein, DT: Decision Tree; LD: Linear Discriminant; LR: Logistic Regression; NB: Naïve Bayes; SVM: support vector machine; ESkNN: Ensemble Subspace kNN; MLP: Multi-Layer Perceptron; BT: Bagged Tree.

In the classification phase, we used the tkNN classifier, which is an iterative and ensemble classifier that achieved high classification performance. To compare the tkNN classifier with other classifiers, we used the 14th feature vector from the EEG violence detection dataset, as it was the most accurate feature vector. The comparative results are presented in Figure 7.

According to Figure 7, the tkNN classifier achieved the highest classification accuracy (99.73%) among the 10 classifiers used. ESkNN, which is also an ensemble classifier like tkNN, reached a classification accuracy of 99.31%. Therefore, tkNN was chosen for the classification phase, and 15 tkNN-based results were generated using the tkNN classifier.

**Figure 8:**
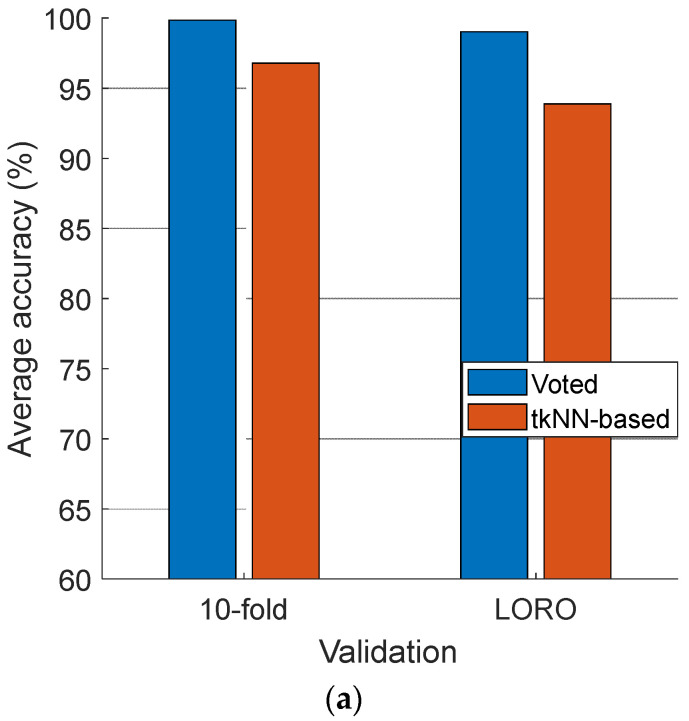

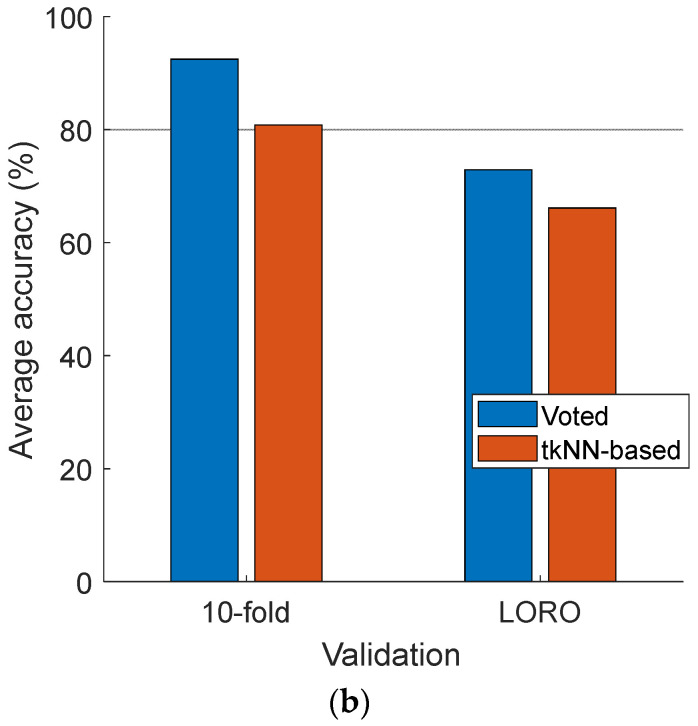
The average classification accuracies of the voted outcomes and the tkNN-based outcomes. (**a**) Violence; (**b**) Stress.

The IMV-based information fusion phase was employed to improve the classification accuracies of tkNN further. By applying IMV, 13 voted outcomes were generated, and the final outcomes for both datasets were these voted outcomes. The average classification accuracies of the tkNN-based and voted outcomes are also shown in Figure 8.

Figure 8 clearly depicts that the voted outcomes have a higher average classification performance than the tkNN-based outcomes in all cases, which clearly highlights the effectiveness of the IMV-based information fusion method.

The proposed ChMinMaxPat-based model was also compared to state-of-the-art (SOTA) models, and the results of the comparison are presented in Table 6.

Table 6 clearly demonstrates that the ChMinMaxPat-based SOXFE model presented achieved a satisfactory ability to classify EEG signals.

The presented ChMinMaxPat-based SOXFE model also generated explainable results for violence detection and stress detection by using the DLob symbolic language. A summary of the DLob strings obtained for violence detection is as follows:

The patterns observed in the DLob sequences suggest that violent videos elicit complex neural responses involving multiple brain regions. The sustained activation of the frontal lobes suggests that participants engage in higher-level cognitive functions to process and regulate their emotional responses to violent stimuli.

Activation of the temporal and occipital lobes emphasizes the processing of auditory and visual information, which is heightened during exposure to violent content. Parietal lobe activation indicates the integration of sensory information and the spatial awareness necessary to interpret the chaotic or fast-paced scenes typical of violent videos.

Analysis of the DLob sequences suggests that exposure to violent videos activates a network of brain regions associated with emotional regulation, sensory processing, and cognitive functions. These findings support the efficacy of using EEG signals and DLob sequences to detect neural patterns associated with the perception of violence.

Understanding these activation patterns will contribute to the development of neural markers for violence detection and reveal how the brain processes violence. Additionally, it sheds light on the examination and treatment of psychiatric cases.

The second dataset was the EEG stress detection dataset, and 15 DLob strings were generated. A summary of these DLob strings is as follows:

The analysis of the DLob sequences shows that exposure to earthquake stress activates many brain regions. These regions are associated with sensory integration, spatial awareness, cognitive processing, and emotional regulation. Sustained activation of the frontal lobes suggests an increased cognitive load, as participants process the stress and plan action. The activation of the frontal right lobe (FR) reflects the need to regulate emotions and impulses during a stressful event.

Activation of the parietal lobe points to the integration of multisensory information and spatial orientation. This is essential for navigating physical spaces safely during an earthquake, avoiding hazards, and coordinating movements. Engagement of the temporal and occipital lobes highlights heightened processing of auditory and visual information. Participants are likely focusing on environmental cues such as the sounds of destruction or visual signs of structural instability.

These patterns suggest activation of neural circuits involved in survival instincts, including fight-or-flight responses. Rapid processing and decision-making are crucial during an earthquake, and this is reflected in the activation patterns observed. Understanding these activation patterns contributes to the development of neural markers for stress detection.

Analysis of the DLob symbol ratios also shows differences in the brain activation patterns between stress and violence. For the frontal lobes (FL and FR), stress shows a higher activation rate. In the FL, this is 29.66%, and in the FR, it is 38.39%, with a total frontal activation rate of 68.05%. In contrast, during violence, the FL activation is 21.96%, and the FR activation is 34.54%, with a total frontal activation of 56.50%. This indicates that the cognitive load and emotional regulation increase during stress, while other brain regions are also activated during violence.

In terms of the temporal lobes (TL and TR), violence exhibits higher relative activation. In stress, the TL activation is 4.58%, and the TR activation is 6.87%, totaling 11.45% temporal activation. In violence, the TL activation is 8.53%, and the TR activation is 5.44%, totaling 13.97% temporal activation. This suggests that violence involves increased processing of auditory information and possibly language or emotional content associated with violent scenes. Under stress, lower temporal lobe activation suggests less emphasis on processing external auditory stimuli and more focus on internal cognitive processes.

The parietal lobes (PL and PR) also show differences. Under stress, the PL activation is 4.15%, and the PR activation is 5.27%, totaling 9.42% parietal activation. In violence, the PL activation is 8.32%, and the PR activation is 5.65%, totaling 13.97% parietal activation. Increased parietal lobe activation during violence suggests enhanced spatial processing and sensory integration required to interpret complex scenes. Lower parietal lobe activation under stress presents less need for spatial awareness during earthquake stress.

For the occipital lobes (OL and OR), violence again shows higher activation. Stress involves OL activation of 5.40% and OR activation of 5.70%, totaling 11.10% occipital activation. Violence involves OL activation of 9.17% and OR activation of 6.40%, totaling 15.57%. Higher occipital lobe activation during violence reflects increased visual processing demands when viewing violent images. Lower occipital activation during stress suggests less pronounced visual processing.

These differences in the DLob patterns suggest predominant frontal activation during stress, with internal processes experienced more intensely. Violence detection also involves internal and frontal processes, but it is more related to external factors than stress.

Additionally, we have demonstrated the connectome diagrams for violence and stress detection in Figure A1 and Figure A2.

The evolution of EEG feature extraction models and symbolic languages from Lobish [32] to DLob [39], and later to TATPat [40] and QuadTPat [41], demonstrates significant advancements in both feature engineering and the interpretability of EEG, providing insights into and experimental results on achieving explainable outcomes through feature engineering. The foundational Lobish model introduced a symbolic approach that translated complex neural signals into interpretable symbols, facilitating early discoveries in explainable EEG analysis. Despite its innovative contribution, Lobish had a limited symbol set which could not adequately represent the right and left hemispheres. Building upon this, DLob added a layer of complexity by incorporating directional symbols to capture the brain activity across tasks better, increasing the model’s capacity for EEG artifact classification using this directional approach. TATPat advanced the DLob framework by introducing a three-node automaton with transition patterns, allowing for the extraction of more complex feature relationships suited to specific applications such as neonatal seizure detection. When combined with Causal Connectivity Theory (CCT), TATPat’s automaton-based approach enabled this model to solve intricate EEG data while utilizing DLob’s symbolic structure for more interpretable outputs. QuadTPat extended this automaton-based structure further, introducing a four-node automaton with quadruple transitions explicitly aiming to improve stress detection. The QuadTPat approach yielded significant findings in stress detection and achieved high classification performance. The progression from Lobish to QuadTPat, with all relevant work published in 2024, underscores that 2024 marks significant advancements in symbolic-language-based explainable methodologies. The development of these models suggests improved accuracy and interpretability for analysis of EEG signals, reflecting the increasing refinement in EEG-based diagnostic systems. In this work, we present the ChMinMaxPat-based SOXFE model, which was tested on both EEG stress detection and EEG violence detection datasets. This research aimed to demonstrate the model’s general classification ability and the interpretability of symbolic-language-based approximations.

The findings, advantages, limitations, and future works are discussed below.

Our findings were as follows:
  - A new ChMinMaxPat-based SOXFE model was presented for classifying EEG signals.
  - The recommended ChMinMaxPat-based SOXFE model was tested on EEG data for both violence and stress detection.
  - The proposed model achieved over 99% accuracy for violence detection and over 70% accuracy for stress detection.
  - ChMinMaxPat extracts 15 feature vectors, and the most informative features are selected using CWNCA.
  - The tkNN classifier, an advanced version of k-nearest neighbors, outperformed nine other classifiers.
  - Information fusion using IMV improved the accuracy, with voted outcomes showing a better performance than that of tkNN.
  - The DLob symbolic language was used to explain the brain activity patterns during violence and stress detection.
  - Our model performed well in 10-fold CV (99.86%) and LORO CV (99.31%) tests for violence detection. For stress detection, it achieved 92.86% with 10-fold CV and 73.30% with LOSO CV. In this regard, the ChMinMaxPat-based SOXFE model presented demonstrated a general classification ability, as it was applied to two separate EEG signal datasets. The recommended ChMinMaxPat-based model achieved over 99% accuracy in violence detection but showed lower accuracy in stress detection with LOSO CV, where the accuracy dropped to around 70%. This difference suggests that some of the extracted features may be effective across multiple tasks, while others may be more task-specific. High performance in violence detection suggests that the extracted features capture neural patterns strongly associated with processing violent stimuli. In contrast, moderate performance in stress detection suggests that these features may not fully capture the neural characteristics associated with stress responses or that stress-related EEG patterns are more variable and less distinct. Moreover, stress has multiple sources, and it is more complex to detect than violence.
  - The DLob arrays/strings generated for both tasks (EEG stress and violence detection) provide insights into the brain regions activated during each type of stimulus. The DLob arrays for violence detection (Table A1) show higher activation of the temporal, parietal, and occipital lobes in addition to the frontal lobes. This widespread activation reflects the complex processing involved in perceiving and responding to intense stimuli, including sensory integration, spatial awareness, and emotional regulation. In contrast, the DLob arrays for stress detection (Table A2) show predominant activation in the frontal lobes, particularly the right frontal lobe (represented by the FR symbol), which is associated with emotional regulation and cognitive processing under stress. The lower activation in the other lobes clearly indicates that stress responses are more internally focused cognitive processes, originating from internal rather than external sensory processing. Additionally, the resulting cortical connectome maps indicate that unique DLob-based patterns can be generated for each condition. These differences highlight that the neural patterns, and hence the features extracted by the presented ChMinMaxPat-based model, are influenced by the specific nature of the task. Features that effectively capture the neural response to intense violence stimuli do not generalize effectively to stress detection due to the different underlying neural mechanisms.
  - Violence triggered more activity in the temporal, parietal, and occipital lobes compared to stress.
  - Connectome diagrams were created to visualize brain activity during violence and stress, highlighting different neural responses.
Our research had the following advantages:
  - The recommended ChMinMaxPat-based SOXFE model achieves over 99% accuracy for violence detection and over 70% for stress detection;
  - It provides clear results using the DLob symbolic language, showing how the brain reacts to violence and stress;
  - The ChMinMaxPat feature extractor introduced generates 15 features, and CWNCA picks the most important ones, improving both accuracy and speed;
  - It adjusts itself during classification and information fusion, boosting the accuracy without complicated settings;
  - Unlike deep learning, the recommended SOXFE model has linear time complexity;
  - It works well with different EEG datasets, making it flexible for various tasks;
  - Its explainable results and connectome diagrams help with understanding brain activity, which is useful in clinical and forensic settings.
Our study had the following limitations:
  - The model was tested on relatively small datasets, which may affect how well it performs on larger, more diverse datasets;
  - While the presented SOXFE model performs very well for violence detection, its accuracy for stress detection is lower, around 70% with LOSO CV.
Future work consists of the following:
  - The recommended ChMinMaxPat-based SOXFE model is planned to be tested on larger, more diverse datasets to improve its robustness by capturing the variability of EEG across individuals and tasks;
  - Feature extraction methods tailored to capturing task-specific neural patterns will be developed to increase the model’s performance across various tasks;
  - Validation of the model on new tasks and datasets will be conducted to gain more findings into the generalizability of the features extracted by ChMinMaxPat.
  - Advanced feature selection techniques prioritizing generalizable features are planned to be implemented to improve the model’s adaptability across tasks;
  - The integration of EEG with other physiological signals or behavioral data will be explored in future research to potentially enhance the accuracy for task-specific applications;
  - The model will be tested in hospitals and clinics to support the detection of mental health conditions;
  - Real-time monitoring of brain activity will be enabled;
  - Next-generation DLob-based EEG translation applications are being planned to broaden the model’s utility in EEG interpretation, with a DLob dictionary potentially being created.

## 5. Conclusions

In this study, we proposed a ChMinMaxPat-based SOXFE model for classifying EEG signals related to violence and stress detection. The presented ChMinMaxPat-based SOXFE model performed well on the violence dataset since our model achieved over 99% accuracy with both 10-fold CV (99.86%) and LOSO CV (99.31%). For stress detection, the presented ChMinMaxPat-based SOXFE model achieved 92.86% with 10-fold CV and 73.30% with LOSO CV. These results showcase the ChMinMaxPat-based SOXFE model’s high classification ability, especially for violence detection.

By using the DLob symbolic language, the model provided explainable insights into brain activation patterns. It revealed that the frontal lobe was the most affected in both violence and stress detection. Stress showcased higher frontal activation (68.05%), while violence activated the temporal, parietal, and occipital lobes more prominently.

The recommended ChMinMaxPat FE function generates 15 feature vectors, the CWNCA selects the most informative ones, and the selected features are classified using the tkNN classifier. Information fusion with IMV further improves the classification accuracy. In the last phase, the interpretable results are created by deploying DLob. This SOXFE model not only achieved high classification performance but also offered interpretable results. Therefore, the model presented is a useful model for both clinical and forensic applications.

Future work should focus on testing the model on larger datasets, improving its accuracy for stress detection, and implementing real-time monitoring capabilities in medical environments.

## Figures and Tables

**Table 1:** The distribution of the EEG violence dataset.

No	Class	Number of EEGs	Number of Records	Number of Participants
0	Control	442	38	8
1	Violence	286	47	6
Total		728	85	14

**Table 2:** Distributions of the EEG stress detection dataset.

No	Class	Number of EEGs	Number of Records	Number of Participants
1	Stress	1757	150	150
2	Control	1882	160	160
Total		3667	310	310

**Table 3:** The used DLob symbols and meaning.

No	Channel	Symbol	Meaning
1–4	AF3, F7, F3, FC5	FL	This represents logical thinking, planning, and decision-making. It is associated with speech production, Broca’s area, which is essential for language articulation and processing. It is involved in sequential thought processes, working memory, and the regulation of voluntary movements. This region also contributes to problem-solving abilities and attention control, facilitating complex cognitive tasks. The left frontal lobe plays a significant role in executive functions, emotional regulation, and the integration of information from other brain regions to form coherent responses.
5	T7	TL	Language comprehension and production are represented here, including Wernicke’s area, vital for understanding spoken and written language. Involved in verbal memory and the processing of semantic information, it contributes to the recognition of words and sentences. This area is essential for meaningful communication and language-related learning processes. The left temporal lobe also participates in auditory processing, allowing for the discrimination of sounds and the interpretation of complex auditory stimuli, including language nuances and emotional tone.
6	P7	PL	Language and mathematical processing are represented in this region. It plays a role in understanding and producing speech, reading, writing, and numerical computations. The left parietal lobe integrates sensory information related to language and assists in tasks requiring attention to detail, logical reasoning, and spatial orientation in linguistic contexts. It is involved in the processing of tactile sensory information, helping to perceive and interpret touch sensations, and contributes to proprioception, the sense of body position in space.
7	O1	OL	Visual information processing from the right visual field is represented here. Involved in recognizing letters, words, and other visual stimuli related to language, the left occipital lobe plays a crucial role in visual perception and interpretation. It contributes to tasks such as reading and the visual recognition of symbols, aiding in language comprehension. This region is essential for processing fine visual details and is involved in the analysis of shape, color, and motion, which are critical for accurate visual recognition and interaction with the environment.
8	O2	OR	Visual information processing from the left visual field is represented here. Involved in recognizing faces, scenes, and the spatial orientation of objects, the right occipital lobe contributes to visual–spatial processing. It aids in depth perception and interpreting visual motion, essential for navigating the environment and recognizing visual patterns. This area is crucial for holistic visual processing, enabling the recognition of complex images and the perception of visual scenes as a whole.
9	P8	PR	It plays a role in recognizing patterns, shapes, and object positions. The right parietal lobe is essential for processing non-verbal cues, spatial orientation, and integrating sensory information to form a coherent perception of the surroundings. It facilitates activities like map reading, spatial reasoning, and the understanding of spatial relationships between objects, which are critical for movement coordination and environmental interaction.
10	T8	TR	Sound processing, including aspects of language and music, is represented. Involved in recognizing faces and objects, the right temporal lobe contributes to the processing of auditory information, particularly the nuances of tone, pitch, and rhythm in music. It is important for memory associated with visual and auditory stimuli and plays a role in interpreting the emotional content of sounds. This region is also involved in the recognition of complex patterns and the retrieval of non-verbal memories.
11–14	FC6, F4, F8, AF4	FR	The right frontal lobe is associated with divergent thinking, emotional expression, and interpreting social cues. It contributes to empathy, understanding others’ emotions, and creative problem-solving. This area is involved in the regulation of behavior, the processing of emotional responses, and the management of attention and motivation.

**Table 4:** The parameters of the methods used.

Method	Parameters
ChMinMaxPat	Input: channel values of each data point Identity generation: minimum and maximum Distance computation: distance of the average value Feature vector generation methods: transition table, channel-based coding, and histogram extraction and concatenation The number of feature vectors generated: 15The length of the features: The number of the channels is 14; therefore, the following was used: 196: f1–f6, f8–f13; 1176: f7, f14; 2352: f15.
CWNCA	Threshold value: 0.99 A variable number of features is selected per the dataset used
tkNN	k: 1–10 Distance: city block, Euclidean, Cosine Weight: inverse, equal The number of outcomes generated: 118 Final outcome selection method: maximum classification accuracy
IMV	Loop range: from 3 to the number of outputs Sorting criteria: classification accuracy Sorting method: descending Voting function: Mode
Greedy	Selection of the most accurate outcome
XAI generation	Number of DLob symbols used: 8 Entropy: information entropy Connectome generation method: symbol transition

**Table 5:** The classification results (%) of the introduced ChMinMaxPat-based model on the EEG violence and stress detection datasets.

Metric	Dataset			
	EEG Violence Detection		EEG Stress Detection	
	10-Fold CV	LORO CV	10-Fold CV	LORO CV
Accuracy	99.86	99.31	92.86	73.30
Sensitivity	99.65	99.65	92.32	69.86
Specificity	100	99.10	93.36	76.57
Geometric mean	99.82	99.37	92.84	73.14

**Table 6:** The comparative results.

Study	Method	Dataset	Accuracy (%)
Shah et al. [37]	EEG temporal–spatial network	1. IDD (14 participants) 2. SEED datasets (15 participants)	1. 5-fold CV: 99.57 2. 60:40: 98.50
Tahira and Vyas [38]	CNN	Physionet EEG dataset	10-fold CV: 99.20
Our method	ChMinMaxPat-based SOXFE model	1. Collected data (286 violence, 442 control) 2. Collected data (1757 stress, 1882 control)	Violence dataset 1. 10-fold CV: 99.86 2. LORO CV: 99.31 Stress dataset 1. 10-fold CV: 92.86 2. LORO CV: 73.30

## Data Availability

The authors are committed to making the data available if requested by the journal.

## Appendix A

In this section, the connectome diagrams created by deploying the selected 15 feature vectors are showcased in Figure A1 and Figure A2, and these cortical connectome diagrams were generated using the DLob strings. These strings are tabulated in Table A1 and Table A2.

**Table A1:** The DLob strings generated for EEG violence detection.

No	DLob String
1	FRPRFRFRFRFLFRTRFRORFRFLFRTLPLFRFRFRFROLFRTR
2	FLFRFRFRFLTRFLTRFLORTLOLFLFLOLTRFLORFLFLPRORFLOLFLOLFRORFLFLFLFRFLTLFLPRFLPLFRPLFLFLFLPLTLFRPLFRFLFRORFRPRPR
3	FRFLFRFRFRPRFRFLFRORTLPLFRTRFRTLFROLFRTRPLOLFRPLPLFR
4	FLFRFLFRFLFRFLFRFLFLFLFLTLFRORFRFLFROLOLFLFRTRFLTLFROLPLORFROLFRFLFLFLOLTLFLFRFRFRFROLFRPLFRPLFRPRFRFLFROLFLFRFLFLFRFLPLFRFRPRFRFLOLFLOR
5	FRFRFRFRFLFLFRFRTLFROLORPLFLORPRTLOLPLOLTLPLOLFLORFR
6	FRTRFROLFRFRFRTLFRFLFRPRFRORTLFLTLFLFRORFRFLFRFRFROLFRFLFRFRFRFLPLFRFRFLFRFRORFRFRTLFLFRFRPLFRTLTLTRFRFRTLOLFRPROROLPLFRFRFLPLTRFRPLFRORPLTLFLPLPLFLPRORFRTRTLTLPLFLFRFRFRFLORTLFRPLFROLORORTLFRFRFLPROLORFLTLFLTLFR
7	FLFLFRFLFRPRFLFLFRTRFRFRFRFRFRTLTLFLFRTRFRFRFLTRFRFLFRPRFLOLFLOLFRFLFRFRFROLFLPLFLTRFRFRFROLFRTRFLFRFRFLFLORFRORFRFRFRTLFRPR
8	PRFRPLFRTRFRTLFROLFRFLFRORFRTLFRPLFRFLFRTRFLORFRPRFRTRFROLFLFLFRFLOLFLFROROL
9	TRFLPRFLPLTLTLFLPRTLOLTLFLTLPLFLTRTLFLTRFLOLTLFLORTLTRFLORFLORTRTLTLOLTROLFLTRFRORFRTRPLFLFLPRFRPRORPLORPLPROROLORFLTLOLORFLFLTLORFRPLFRTLTRTRFLOLFLTLFLPLPLFLTRPLFLPROLFLFLOLFL
10	TRFRPRFRORFROLFRPLFRFLFRTLFRPLFRPRFRORFRFLFRPLOLTLPLTRFLTRFRTLFRFLOLFLFROLFLPRFLFLOLPLFLFLFR
11	FRFRFRTLFRFLFRTRFRFLFROLFRPLFRTLFRFLFRPRFRFLPLFLFRFL
12	FRFRFRFRFRPLPLOLFLFLFRFRFROLFROLTLPL
13	FRFRFLFRFLFRPRFRFLFRPLFRFLFRORFRTLFROLOLFLPRFLFRFLOLFLFRFLFLFLPRFLOLOROLFLPLFLPLFLFLOLFRFRPLORPLFLPLFLPLFROLFLFRPLPLFLOLFROLPROLFRPR
14	TLFLFRFRFLFRPRFLPRTLFROLORFRTLFROLFRFLFRFRTRTRFLORFRFLFRTLFRFRFLPRFRFRPLPLFRTRFRTRFRORFLFLFRPRORPLTLPRFRPLFLTLTLFRFRPLPRPLORPLFLPLFROLFLPRFRTLFRTLFLFLFRFLFRFRFLFRORPRFRORFRFRFLFRTLPRFROLOLORFRFRFLORFRFRFRFRORPLFRPLOLFRFRFROLTRTLPRFRFLFLTLFRPLFRFRTLOROLFLFRFLOLOLFRFLFRPLORFRTLFLOLPLOLTRFLFRFLFLOLTRPLFLFLPLFRFLOLFLOLFRPRFRPLTLFL
15	PLFRTLFRFRFLFLFLFRFRFLFRFRTRPRTLFRTRFRPRORFRPLTLFLPLFRFLFRFRFRTLFRPRFRFLTLFLFRFRTRTLFLTROLFRFLTRFROLFLFLFRFRFRFRFRORFRFRFRTROLFLPRFRFROLPLFRFRTRFLOLFRORTLTLFRTRTLFRPRFLPRORFLFRFROLFRTLFLFRFRTRFRFLFRFLFRTLFLFRPLFLFRORFROLFROLFROLFRFRFRPRTLFLPRFRTLOLPRFRFRFR

**Table A2:** The DLob strings generated for EEG stress detection.

No	DLob String
1	FRFLFRFRFROLFRTRFRFRFRFRFRFLFRTRFROROLFLFRORFRFRFRPRFRFLFRTRFROLFRFRFRTLFLFRFRPLFRPLFLPRFRFRFRFLFRPRFLTRFLFLFLTRFLFRFLFLORFLFRFLFLPROLFLOLFLTRFLFRTLFLFLFLFRFRFRTRPRFLFRFLOLOLFRFLFROLTRFLPLORFLFRFRFLFRFLORFLFLFLFLFLOLOLFRFRFRPRPRFLORFLTRFRFRFLFLFLTLOLTLORFLOLFLPLFRFRTLPRFLOLFRORFRFLPRTRFLTRFLFRPLOLOLFLPLTLPROLFRFLFRFLFRFLFLFLPLORTLPRFLFRORTRFLTLFRFROLFLTLORFRFLFRFLOLFLTRFLTLPRTLTRORPRFRTROLFLFRFLFLFRFLPLTLFLFRPRFRTRFRFRFLORTRFRORFLFRORFR
2	FLFRFLFRFLFRORFLFLFLPLFRFRFRFLFRFLTRFRFLOLFLFLTRTLFRPRFRFLPLFRFRFRPRTRORTRPLORFRPLFLFLTRFLPLTLFLFLORFLPRFRFLPLTLFRFLOROLFLORFLOLORFLORFLPRFLFLFLTLFLTRFLFLFROLFLFLFLFRFRFLTRPRFRPLFLFLFLFRFROLFRFRTLFLOLFLFRTRFLFRFLFRTROLTLFRFLTLPRPLFLFROLTLFLORPLFRFLFRPRTRFRFLORTRFLTLTLFRFLORFRFRFLTLTRPLFRFLFRFLOLOLFLFRTROLFROLTRORTRFRTRPLTRTROLFLPRFLFLORFRFLFRTRPRPRPRTRFRFLFRFRFLFRPLORFLPRFRPLFRFRFROLFLFRFLORFRTRTRFLFLFRORTLOLPRFRFRFLFRFLFLFR
3	FRFLFRFRFRFRTRFROLORFLTLFLFLFLFLFRTRFLFLFRFRFRTRFRFRFRTRFRFLFRFLFRFRFRPRFRFRFRPLPRTRFLFLFRFLFRFLFLFRFROLFRFRFRFLFRPRFLTRFLPRFLOLFRFLFLTLTRFLFLORFRPRFRFRFRFRORPRFLPLFLOLFRPLFLORFLFRFLFLFLFRFLFLPRFLFLFLFLFRPRTLFLOLTLFRTRFLTLPRFLPRTRFLPLTLFLFRPLPLPRORFRFRFLOLTRORPRFLTRFLFLFLFLFRFLFRFRFR
4	FLFRFLFRFLFRFLFRFLFLFLFLPLFRFRFRFRFLFLTRFLFRFLFRFLOLPRORTRFLFLFLFRFLFRFRFLFRFLTRFRFLOLFRORFRFLPRPRPRORORFRTRTLFRPLORFRFRFRFRTLPRFRFLFLTRFRTRFLOROLPROLOLFLTRFLFRORTROROLFRFL
5	FRFRFRFRFLFLFRFRFLFLFRFLFRFRPRTRFRFROLORFLFRFLPRFLTRFRTRFROLPRFRFRFLTLFLFLOROLFRFLOLFLFLFRFLFLFRFLFRFRFLFRORORFLFRFRFLFRTRFRFRFRFLFRFLFRFLFRTRFRFLFRFRFRFLPRFRPRTLTLOLFLFRFRFRORTRFLOLFRFRPLFRFLPRFRFRFRFLFLFRFLFROLFRFR
6	FRFLFRTLFRFLFRORFRFLFRFRFROLTRFLPLPLTRTLFLPLFLFLFRFLFRFRFRFLFRFLFRFRFRPRPROLFLTLFRTRFRFLFRORFLFRFLFRFLFRFRTLFRPLFLFRFROLFRPRFROLTRPLFRFLFRORORPLPRTLFLFRFROLFLTLFLFLFRTLFLPLFRFRFLFROLFRFRPLORFLFRFROLPLFLOLORFLFRPRTRFROLPRFRFRFRTRFRFLFLPRFLFRFRFL
7	FLFRFLFRFRFRFLFLFRFRFLFRFLFLORFLPLFRFLFRFRPRFLTRFRFRFRFRFLFRFRFRFRFRFLPLFRPLTLFLFLFRFRTRFLFLFRFRTRFLFRFLFRTRTLFRORFRFLFLFLFLFRPRFLFLFRTRFRFRFRFRORPRFLFLFRFLFRFRFLFRFRFRTLFLFRFLFLOLFRFRFRORFRTRFLFLOROLPRFLFLTLORFLFROLFLFRFRFROLORFLTRFRFLFROLFLPRFRPRFRTRFLOROLFRFLFRFRORFLOLFRFRFRFRFROLFLFRFRFLORORFLFLFRFLFRTLFLFLFLFLFRFLFROLFLFLTRFLFRFRFRFRFROLFLFLFLFLFRPRFLTRFRFLFRTRFRFLTRORFLOLFRFRFRFLFRFRTLFRFRFLFLORFRPLFRPRFRFLFLFRTLFLFRFLTRTLFLOLFRTRTRFRFRFLFLFRFRORFLTLFRFLFRFRFRPLFRORFRFLFLPLFRORFRTRFLFLFROROLORFRTLFROLFLTLFRFRFRFLFLORFRFLTLFLFRORFRTRFRPLTRFRPRFRFRPRFLFLFRFR
8	TRFRFLFRFLFRPRFRPLFRPRFRTRFRFLFROLFRFLFRORFRFRFRTLFRFRFLFLFROLFRFLFRFRFRFRORFRFRFLFRFLFRFRFRFLPRFLFLFROLTRFRFLTRTRFLORFRPLFRFRFLFLFLFRFLFRORTLFRFRTRFLORTRFLORTRFRFLFLORFLPRFLFL
9	TRFLTRFLFRPLFLPRPRTLTRORTRFLTRFRFLTLFRPROLFRTRPLFLFLPLFLTRPRTROLFLFLFLPLPROLPRFLFRFLFLFRTRTLOLTRFRTRFLFRFRORFRPLFLFRFRFLPLFLFRTLTLTLTLFLFRORFLFRFLOLFLPRFLFLFLFLFRFRFRFLFRFLPRFRFRTRFRFRFLPLFLOLFRPLFRFLPRFLFRFLFLTRFLOLOLFLFRFRPLFRFLFLFLORFLFLFLFLPLFRFLFRFRFRFROLFRFLFRPRFRFLORFR
10	FLFRFLFRFLFRTRFRPRFRTRFRPRFROLFRTRFRFLFRPLFROLORFRFLFRFRFRFRFLFLORFRTLFRFRFRFRFRFLFLFLFLFLFRFLFLFRFRPLFRTLFRFRFRORFRFLFRFRTRFLFRFLFRTRFLTRFRFRFRFLFLFRPRFRORTLFRFLFLFLFL
11	FRTLFRFLFRFLFRFLFRFLFRTLFRFRFRFLFLTLFRFLFRFRFRTLFRTRFROLFRFRFRPRFROLFRFLFRFLFRFRFRPLFRPRFLPRFROLFRFLFRFLPLFLFRFLFLPRFRORPLFRFRFRPLPRFLFRFRFRORFLFLFROLTRFLFLFRFLPLFLPRFRFRTRORPLOLPRPRFLFLFLFLORPRFRFRFRFRORFRFLFLTRFRPLFLTLOLFRFRFR
12	FRFRFLFLFRFRFLFLFRFLFRFRFRFROLFLFRFLFRFRFRFRFRFLFRFLFRTRFRFRPRFLOLORFROR
13	FLFLFLFRFLFRFLFRFLFRORFRFRFRTLFRFLFRTLFLFRFLFLFRPRFRFRFRPLFRFLFLTRFRFLFRFLFLORFRFLFLFLFRFRFROLFRFLTLPRFRPLFRTRFLTRFRFLFRFLFLFLFLORFLPRFRFLFRTLFLPLFRFLOL
14	TRFLFLFRTLFRFLFRFLFRTRFRTRORFRTLORFRPRTLTRFLFLFLPRFRFRFRFRFRTRFRPRFLOLFRFRFRFLFRFLFLTRTLPLFRFRFLFLFRTLFRFLFLFRFLORTLTLFRTRFRFLFLPLTLTRFLFLPLTRPLORFRFLFRFRFLFROLFRFRFLFLFLFLOLORFLFRFLFRFLFRFLFLORFRFRFRFRORFRFRFRORTRFRPLFRFRFRFLFRFLFLFLFRFLTLFLFRFRTRFLFLFRFLPRFRFRPLOLFRFLFRFRFRFRFLFRFRFLFRFRFLFRFRFLFLFLFRFRFROLTRFRPRTRPROLFRPRFRFRFLFRFRFRFRFRFLFLFRPLFRFRFLTLFRFRTLORFRPRFRFLFRFRORFRFRFRFLFLFRPLFLFROLFRORTRFRFLFRFRPROLFRPLFRFLTLPLFRPRFRPLFLPRFRFRPRPLFRTRFRFRFRFRFRTRFRFLFLFRFRFRFLTRFLFLFLFRFLFLFRFLFLFLORFLORFRFRFROLFLFRFLORFRFLFRTLPLFR
15	TRFLTRFLFLFRFRTLFLFRTRFRPRFRFLFLFLFRTRFLFRFRTRFRTRTLFLFLTRPLFROLFLFLTRFRTRFROLORFRFRPRTLFLFRPRFLTLFRFRFRFLFRFLFRORFRFRFLPLFLFLFLFLFLFRFRTRORORFRORFRFLFLFLFRFLFRFRFRFRTLFRFRFRFRFRFLFLPLFRFRFLFRFLFLOLFROLFRORFRORFRTLFRTLFRFLFRFLFRPLFRFRFLTLFRPRFRFLORFLFRFLFRFRORFLFRFRFRFRFRPRFRFLFRTRFRFRFLFLFLFLFLFRFLTRFRTRFRTLFLFRFLTRPRFLTLFLFRFLFRFRFLFRFRFRFLFRFLFRFLPRFRPRFRFLFRPLFRFRFROLTRFRFLOLFRFLFRFLFRFRFLFRORFROLFLPRFLFRFRTRPLFRPLFROLFRPLFRFLFRFRTLFLFLFLFRFLFRFRPROLFROLFRFLFRFLFRFRFLTRFRTLFLTLFLTLTLFRFRFRFROLFLFRORTLFRFRFLOROLFRPLFROLFRFROLFRFLFRFLFRFRFLFRFRFLFRFRFRFRFRFLFLFLFLFRTRPRFRPLFLFRFRFRFROLFLFLOLFRPRPLFRFLFLFLFLFRORFRFLFLFRFRTRFLFRFRPLFLFRORTLORFRFLFRFLFRPLTLORFRFLFL
